# Applicability of ISNT rule to retinal nerve fiber layer thickness by SD-OCT in normal subjects

**DOI:** 10.22336/rjo.2024.46

**Published:** 2024

**Authors:** Sujata Lakhtakia, Anamika Dwivedi, Aashi Jain, Dhirendra Kumar Pandey, Pooja Singh, Divya Tripathi

**Affiliations:** 1 Department of Ophthalmology, SS Medical College, Rewa, MP, India; 2 Department of Ophthalmology, NSCB Medical College, Jabalpur, MP, India; 3 LV Prasad Eye Institute, Hyderabad, AP, India

**Keywords:** retinal nerve fibre layer, neuro retinal rim, ISNT rule, SD-OCT, normal subjects, ISNT = Inferior>, Superior>, Nasal>, Temporal, NRR = Neuro Retinal Rim, RNFL = Retinal Nerve Fibre Layer, SD-OCT = Spectral Domain Optical Coherence Tomography, IST = Inferior>, Superior>, Temporal, IS = Inferior>, Superior

## Abstract

**Aim:**

The usefulness of the Inferior>Superior>Nasal>Temporal (ISNT) rule for the neuroretinal rim (NRR) has been widely used in differentiating normal eyes from glaucoma, but only a few studies have assessed whether this classical ISNT rule applies to the retinal nerve fibre layer (RNFL). This study aimed to determine the applicability of the ISNT rule for the peripapillary RNFL thickness in normal eyes using Spectral Domain Optical Coherence Tomography (SD-OCT) and assess if variants of the ISNT rule apply.

**Methods:**

A cross-sectional study was conducted on 120 eyes of 62 healthy subjects who fulfilled the study criteria. Peripapillary RNFL thickness was measured by OCT scan and each eye’s global, superior, temporal, nasal, and inferior quadrant thickness was noted. The values obtained were analyzed to determine the percentage of eyes obeying the ISNT rule and its variants.

**Results:**

The ISNT rule for RNFL thickness was applicable for normal subjects in only 53.33% of cases. Removing the nasal quadrant from analysis increased the number of eyes obeying the IST rule to 71.66%. Further exclusion of the temporal quadrant showed almost the same results (75%) for the IS rule.

**Conclusion:**

The ISNT rule for RNFL thickness could be validated in only 53.33% of normal individuals. Though documented as useful for NRR during ophthalmoscopy in glaucoma diagnosis, the ISNT rule did not apply to the quadrant values on RNFL on SD-OCT examination.

## Introduction

The neuroretinal rim (NRR) comprises axons of retinal ganglion cells and forms an important component of the optic nerve head. Jonas et al. reported that in normal individuals, the NRR exhibits a characteristic configuration with the maximum thickness being in the inferior quadrant (I), followed by superior (S), nasal (N), and temporal (T) [1]. This specific pattern of the neuroretinal rim was termed the “ISNT rule”. Since retinal nerve fibers form the NRR, it can be logically assumed that the retinal nerve fibre layer (RNFL) would also follow the same pattern. Some histological studies in nonglaucomatous eyes have also shown that the RNFL exhibits a similar thickness pattern, with the inferior quadrant being the thickest, followed by the superior and the nasal, and the temporal quadrant being the thinnest [2,3].

Since thinning of both NRR and RNFL, particularly in the superior and inferior quadrants, are hallmark features of glaucomatous optic neuropathy [4,5], any deviation from the ISNT rule for NRR and RNFL thickness may be an early indicator of glaucomatous structural change.

Qualitative assessment of the neuroretinal rim in terms of its quadrant-wise thickness is widely used for clinically differentiating between glaucomatous and non-glaucomatous discs. This is done by ophthalmoscopy but has the disadvantage of being a subjective clinical sign. RNFL thickness on the other hand is assessed by the spectral domain optical coherence tomography (SD-OCT), which provides objective, quantifiable, and reproducible measurements.

Only a few studies have evaluated the applicability of the ISNT rule to RNFL thickness in normal individuals, but their results have been equivocal.

Hence, this study was conducted to determine if the ISNT rule applies to the RNFL thickness using SDOCT, in normal eyes, and to see if variants of the ISNT rule applied.

## Methods

This cross-sectional study was duly approved by the institutional ethics committee and carried out at the Glaucoma Clinic of a Tertiary Health Centre in central India. Sixty-two subjects who satisfied the study criteria of being 18 years or more of age, having no ocular abnormality except senile cataract, best corrected visual acuity of 20/40 or better, refractive error not exceeding +3D and absence of glaucomatous optic neuropathy were included in the study. Individuals with any condition that could affect RNFL thickness and those with OCT scan signal strength of <6/10 were excluded.

After enrolment, all study subjects underwent a comprehensive ophthalmic examination, which included assessment of best corrected visual acuity, refraction, anterior and posterior segment evaluation, gonioscopy, applanation tonometry, and visual field analysis.

Peripapillary RNFL thickness was measured by OCT scan, using Cirrus HD OCT Model 500 (Carl Zeiss Meditec, Inc. Dublin, CA, USA). RNFL analysis was derived from the optic disc cube 200x200 scan protocol, which provides information on the circumpapillary RNFL thickness.

Each patient was scanned three times and scans with a signal strength of six or more were used for analysis. Each eye’s global, superior, temporal, nasal, and inferior RNFL thickness was recorded. If two quadrants of an eye exhibited equal thickness values, then the eye was excluded from the analysis.

The sample population was described via descriptive statistics including numbers (percentages) and mean (**+/-** standard deviation SD). The chi-square test was used for statistical analysis and a p-value less than 0.05 was considered statistically significant.

## Results

The present study was conducted on 120 eyes of 62 healthy subjects. The age of the patients varied from 31 to 76 years with a mean age of 45+14.31 years. The gender profile showed an almost equal distribution of male and female patients with a male-to-female ratio of 0.9:1.0.

The clinical parameters of the study subjects are exhibited in **Table 1**. The mean global RNFL thickness was 98.6±12.8 µm. Though the mean quadrant-wise RNFL thickness values followed the ISNT rule, the same did not hold for all individual eyes. The inferior quadrant RNFL was found to be the thickest in most eyes (n=90; 75%) but it was observed that 30 (25%) eyes showed maximum thickness in the superior quadrant. The temporal quadrant RNFL was the thinnest in 87 (72.5%) eyes but the thinnest nasal RNFL quadrant was exhibited in 33 eyes (27.5%). We also observed that the RNFL in the nasal quadrant was thicker than the superior in 12 (10%) eyes.

**Table 1:** Clinical parameters of study subjects

PARAMETER	MEAN VALUE
Spherical equivalent	-0.9+1.4
Intraocular pressure	14.5±2.1 mmHg
Visual field Mean deviation	-0.3±0.6
Visual field Pattern standard deviation	0.24±0.5
Global RNFL thickness	98.6±12.8 μm
Superior RNFL thickness	120.1±15.4 μm
Inferior RNFL thickness	131.4±14.8 μm
Nasal RNFL thickness	73.6±10.7 μm
Temporal RNFL thickness	69.5±9.2 μm

Analyzing the number of eyes following the ISNT rule for RNFL thickness, we found that this rule applied only in 53.33% of eyes. However, it was seen that when the nasal quadrant was not considered, and only the superior, inferior, and temporal quadrants were assessed, the number of eyes obeying the IST rule increased to 71.66% (n=86). Furthermore, the temporal quadrant exclusion from the analysis showed a marginal increase in the applicability of the IS rule to 90 (75%) eyes as shown in **Table 2**.

**Table 2:** Applicability of ISNT rule and its variants

ISNT Rule	Number of eyes (%)
I>S>N>T	64 (53.33%)
I>S>T	86 (71.66%)
I>S	90 (75%)
T thinnest	87 (72.5%)

## Discussion

The ISNT rule was initially developed for the neuroretinal rim of the optic disc by Jonas JB et al. [1], and this was attributed to the slightly vertically oval disc having a slightly horizontally oval cup. Subsequently, histological studies on human eyes documented a similar pattern of I>S>N>T for the retinal nerve fibre layer [2,3]. The SD-OCT development allows us to test Jonas’s theory that the number of nerve fibers reaching each quadrant affects the rim area. Hence, the current study assessed and compared the RNFL thickness in the four quadrants to ascertain the applicability of the ISNT rule to the RNFL.

The major finding of this study was that the ISNT rule for RNFL thickness could be validated in only 53.33% of eyes. Although the mean RNFL values for the entire study cohort followed the ISNT rule for individual subjects, this applied to only slightly more than half of the eyes (**Table 2**). This was per other studies in which the percentage of individuals not following the ISNT rule for RNFL thickness varied from 42% to 55% [6-10]. The reason attributed to this observation in the present study is that the temporal RNFL was thicker than the nasal in 27.5% and the superior RNFL was thicker than the inferior in 25% eyes.

Dave P et al. compared the applicability of the ISNT rule in Indians, between normal individuals and those with early glaucoma, and documented that 55% of normal individuals followed this, which is quite like the results observed in the present study [6]. SD-OCT studies done by Alasil T et al. and Poon LY et al. on normal eyes found that only 42% and 43.8% respectively followed the ISNT rule [8,9]. The population selection could explain this difference from our study, as ours was done only on Indians. Another study, by Pradhan Z et al., which found a lower percentage (47.1%) of ISNT applicability in RNFL thickness, used the Heidelberg retinal tomogram that probably overestimates the nasal rim due to the presence of large retinal veins and that could explain the difference in the values measured [7].

Certain studies have suggested that the nasal rim is excluded from the morphometric disc analysis as it is the last to be affected in glaucoma and because of the presence of the retinal vein, the borders of the optic cup are hard to determine in the nasal quadrant [11,12].

Since the validity of the ISNT rule was violated mostly because of the thicker nasal RNFL, it would logically follow that if the nasal quadrant is excluded from the ISNT rule, this rule would be more widely applicable to the general population.

When we applied this to the subjects in our study, we found that the validity of the IST and IS rules increased to 71.66% and 75% respectively.

Studies by Alasil T et al. and Poon LY et al. that evaluated the applicability of the ISNT rule variants on RNFL thickness using Spectralis OCT and Stratus OCT found the IST rule to be valid in only 60% and 58.7% respectively [8,9]. In contrast to these two studies, using SD-OCT, Arvind H et al. reported that after excluding the nasal quadrant from the analysis, the IST rule was valid in 70.9% and the IS in 71.8% [11].

The results from the present study show that the applicability of the ISNT rule increased, as we removed the quadrants, with an increasing trend (53.33% for ISNT, 71.66% for IST, 75% for IS). However, since the percentage of individuals for whom the IST and IS rules were valid, was significantly (p<0.003; p<0.0001 respectively) more than that for ISNT, using both IST and IS for differentiating between normal and glaucomatous discs could be a better alternative.

Our study had certain limitations, the foremost being that it was done on Indian subjects and hence the results cannot be generalized for all ethnicities. Furthermore, RNFL thickness was assessed in four quadrants, so focal losses within the quadrant may have been overlooked. Also, the correlation of these findings with the NRR of the optic disc was not done, which could have been more decisive, since it is the RNFL that forms the NRR as it exits the eye as the optic nerve.

## Conclusion

In conclusion, the ISNT rule for RNFL thickness could be validated in only 53.33% of normal subjects. Additionally, the number of eyes conforming to the IST and IS rules was greater, thus implying that they can be better predictors of glaucomatous damage than the ISNT rule. In addition, the findings from the present study call to attention that a rule applicable to a gross population may not be valid for all persons because of individual variations.
